# Evaluation of *Lippia scaberrima* Sond. and *Aspalathus linearis* (Burm.f.) R. Dahlgren extracts on human CYP enzymes and gold nanoparticle synthesis: implications for drug metabolism and cytotoxicity

**DOI:** 10.1186/s12906-024-04439-9

**Published:** 2024-04-05

**Authors:** Anna-Mari Kok, Risto Juvonen, Markku Pasanen, Vusani Mandiwana, Michel Lonji Kalombo, Suprakas Sinha Ray, Rirhandzu Rikhotso-Mbungela, Namrita Lall

**Affiliations:** 1 Department of Plant and Soil Sciences, University of Pretoria, Pretoria, 0002 South Africa; 2 Research Fellow, South African International Maritime Institute (SAIMI), Nelson Mandela University, Gqeberha, 6019 South Africa; 3 School of Pharmacy, Faculty of Health Sciences, University of Eastern Finland, 70210 Kuopio, Finland; 4 Nanostructures and Advanced Materials, DSI-CSIR Nanotechnology Innovation Centre. Council for Scientific and Industrial Research, Pretoria, 0001 South Africa; 5 Centre for Nanostructures and Advanced Materials, DSI-CSIR Nanotechnology Innovation Centre, Council for Scientific and Industrial Research, Pretoria, 0002 South Africa; 6 School of Natural Resources, University of Missouri, Columbia, MO USA; 7 College of Pharmacy, JSS Academy of Higher Education and Research, Mysuru, Karnataka India; 8 Senior Research Fellow, Bio-Tech R&D Institute, University of the West Indies, Kingston, Jamaica

**Keywords:** *Aspalathus linearis*, Cytochrome P450, Cytotoxicity, *Lippia scaberrima*, Nanoparticles

## Abstract

**Background:**

Metabolism is an important component of the kinetic characteristics of herbal constituents, and it often determines the internal dose and concentration of these effective constituents at the target site. The metabolic profile of plant extracts and pure compounds need to be determined for any possible herb-drug metabolic interactions that might occur.

**Methods:**

Various concentrations of the essential oil of *Lippia scaberrima*, the ethanolic extract of *Lippia scaberrima* alone and their combinations with fermented and unfermented *Aspalathus linearis* extract were used to determine the inhibitory potential on placental, microsomal and recombinant human hepatic Cytochrome P450 enzymes. Furthermore, the study investigated the synthesis and characterization of gold nanoparticles from the ethanolic extract of *Lippia scaberrima* as a lead sample. Confirmation and characterization of the synthesized gold nanoparticles were conducted through various methods. Additionally, the cytotoxic properties of the ethanolic extract of *Lippia scaberrima* were compared with the gold nanoparticles synthesized from *Lippia scaberrima* using gum arabic as a capping agent.

**Results:**

All the samples showed varying levels of CYP inhibition. The most potent inhibition took place for CYP2C19 and CYP1B1 with 50% inhibitory concentration (IC_50_) values of less than 0.05 µg/L for the essential oil tested and IC_50_-values between 0.05 µg/L-1 µg/L for all the other combinations and extracts tested, respectively. For both CYP1A2 and CYP2D6 the IC_50_-values for the essential oil, the extracts and combinations were found in the range of 1 – 10 µg/L. The majority of the IC_50_ values found were higher than 10 µg/L and, therefore, were found to have no inhibition against the CYP enzymes tested.

**Conclusion:**

Therefore, the essential oil of *Lippia scaberrima*, the ethanolic extract of *Lippia scaberrima* alone and their combinations with *Aspalathus linearis* do not possess any clinically significant CYP interaction potential and may be further investigated for their adjuvant potential for use in the tuberculosis treatment regimen. Furthermore, it was shown that the cytotoxic potential of the *Lippia scaberrima* gold nanoparticles was reduced by twofold when compared to the ethanolic extract of *Lippia scaberrima*.

**Supplementary Information:**

The online version contains supplementary material available at 10.1186/s12906-024-04439-9.

## Introduction

The value and importance of medicinal plants are still essential today, especially in developing countries where access to primary healthcare is limited. Many medicinal products are derived from these plants as pure compounds or standardized extracts and are used to alleviate many symptoms and diseases. Fermented and unfermented *Aspalathus linearis* (Burm.f.) R. Dahlgren (*A. linearis*) have extensively been investigated, especially regarding their antioxidant potential, as stated by Joubert et al. [1]. Minimal research has been conducted on *Lippia scaberrima* Sond. (*L. scaberrima*), with most of the work directed towards its essential oil extract. No extensive data are available on the pharmacokinetic properties of both *L. scaberrima* and *A. linearis* in humans. In addition to being metabolized, *L. scaberrima* and *A. linearis* may cause herb-drug interactions through the induction or inhibition of CYP enzymes found in the liver and extrahepatic organs of the body such as the placenta, kidneys, adrenal glands and gastrointestinal tract [2]. The metabolic profile of plant extracts and pure compounds are very important to determine the potential of any future herb-drug metabolic interactions that might occur.

Nanoparticle-based therapeutics have been approved for various treatments and ailments such as cancer and infectious diseases [3, 4]. Many applications using silver nanoparticles have been used for drug development and delivery by using the body’s natural mechanisms to fight against infections and disease. The chemical synthesis of nanoparticles may take a short duration but can be toxic and have hazardous by-products. Thus, there is an ever-increasing demand for the synthesis of green nanotechnology, which may present less hazardous by-products and lowered toxicity profiles [5]. There are several approaches to synthesizing nanoparticles using biological methods such as bacteria, fungi, and plants [6, 7]. Nanoparticles have distinct properties, such as larger surface-to-volume ratios. They can introduce new or improved properties based on size, distribution and morphology, ultimately leading to improved therapeutic usage for infections such as tuberculosis and cancer [8]. Many different types of nanoparticles exist: copper, zinc, and titanium, as well as magnesium, silver and gold [9–11]. Gold nanoparticles are reported to be used for the treatment of rheumatism, nephrotoxicity as well as cancer [12]. In addition, many plant extracts have been utilized as stabilizers and capping agents to produce nanoparticles [13–15]. Various plant extracts have shown good antibacterial properties but, in many cases, found to be very cytotoxic and, therefore, deemed unsuitable for further use in studies. Many nanoparticles formed from the use of medicinal plant extracts have shown their ability to reduce the toxicity profile of the plant extract in use but also seem to increase their therapeutic potential, be it antibacterial or even anthelmintic, as illustrated by Kar et al. [12].

In the current study the inhibitory potential of the essential oil of *L. scaberrima*, *L. scaberrima* alone and the combinations with fermented and unfermented *A. linearis* were studied against placental, microsomal and recombinant CYP enzymes. A previous study conducted on the cytotoxicity of *L. scaberrima* against liver hepatocellular carcinoma (HepG2) cells by Reid et al. [16] indicated that this extract showed a cytotoxic effect with a 50% inhibitory concentration (IC_50_) of 109.20 ± 8.05 μg/mL. To be able to optimally use this extract for all its noted benefits and to further investigate its adjuvant properties it was decided to investigate whether synthesis of gold nanoparticles from *L. scaberrima* using gum arabic as the capping agent could lower the cytotoxic potential of the ethanolic extract. This is the first assessment of the effects of the essential oil of *L. scaberrima*, the ethanolic extract of *L. scaberrima* and their combinations with fermented and unfermented *A. linearis* against a range of important human cytochrome P450 enzymes.

## Materials and methods

### Cell lines, chemicals and reagents

For the CYP inhibitory studies, the following chemicals and reagents were used: Coumarin and 7-hydroxycoumarin were obtained from Sigma-Aldrich (St. Louis, MO, USA). The synthesis and purity of TFD024 (3-(3-methoxyphenyl)-6-methoxycoumarin), OCA349 (3-(4-trifluoromethylphenyl)-6-methoxycoumarin), TFD008_1 (3-(4-phenylacetate)-6-chlorocoumarin, TFD032 (3-(3-methoxyphenyl)coumarin), TFD023 (3-(4-phenyl)-7-methoxycoumarin) and OCA369 (3-(3-benzyloxo)phenyl-7-methoxycoumarin) are described in previously published literature [17–19], Tris–HCl, magnesium chloride (MgCl_2_), MnCl_2_, isocitric acid, isocitric acid dehydrogenase, Glycin, NaOH and trichloroacetic acid (TCA) were all bought from Sigma-Aldrich (Steineim, Germany). Nicotinamide adenine dinucleotide phosphate (NADP^+^) was bought from Roche Diagnostics (Mannheim, Germany). The NADPH regenerative system consisted of 1.12 mM NADP^+^, 12.5 mM MgCl_2_, 12.5 MnCl_2_, 16.8 mM isocitric acid, 0.056 mM KCl and 15 U isocitric acid dehrydogenase in 188 mM Tris–HCl buffer at pH 7.4. cDNA expressed human wild-type CYPs (CYP1A1, CYP1A2, CYP2A6, CYP3A4, CYP1B1, CYP2C19 and CYP2D6) were obtained from BD Biosciences (Bedford, MA). The human liver used during the current study were obtained from University of Oulu Hospital as excess from kidney transplantation donors. The excess tissue collection was approved by the Ethics committee of the Medical Faculty of the University of Oulu (Doc 01–38; 1 June 2000). The liver samples were surgically excised and transferred to ice, where they were cut and snap frozen within liquid nitrogen and stored at 80 C until the final microsomal preparation. The liver microsomes were prepared as described by Lang et al. [20]. Determining the microsomal protein concentration was done by using the Bradford method. The placental microsomes used during the current study came from a stock previously prepared according to Huuskonen et al. [21]. The microsomes were prepared from both non-smoking and smoking mothers.

For the gold nanoparticle synthesis, the Gold (III) chloride trihydrate (HAuCl_4_.3H_2_O), *N-*Acetyl-L-Cysteine, Bovine Serum Albumin (BSA) and Sodium Chloride (NaCl) were all obtained from Sigma-Aldrich (St. Louis, MO, USA).

For the cytotoxicity analysis The HepG2 cell line (HB-8065) was obtained from Separations Scientific (Roodepoort, South Africa). Cell culture materials and reagents such as Fetal Bovine Serum (FBS), DMEM media and antibiotics were supplied by Highveld Biological). PrestoBlue was purchased from Thermo Fisher (Carlsbad, CA, USA).

### Plant material, collection and extraction

*Lippia scaberrima* was collected during the autumn month of May in 2015 from the Kopela community situated near Delareyville in the North West Province, South Africa. The plant was identified, and a voucher specimen was deposited at the H. G. W. J Schweickerdt herbarium, University of Pretoria (PRU) (Table 1). Unfermented and fermented *A. linearis* ground leaf material were kindly donated by Rooibos Limited, Clanwilliam, Western Cape, South Africa and a voucher specimen deposited. The stem, flowers and leaves (aerial parts) of *L. scaberrima* were ground to a uniform size of 0.2 mm. The ground plant material from both plants were extracted with ethanol at a ratio of 1:10 (weight: volume) and macerated for a period of 72 h. The ethanolic extracts were then filtered, and the plant material extracted with fresh ethanol at a ratio of 1:5 for a further 48 h, followed by filtration. All the filtrates were dried under reduced pressure using a rotary evaporator. The three extracts were stored in airtight containers at 4 °C until further use. Gas chromatography-mass spectrometry (GC–MS) and ultra-performance liquid chromatography- quantitative time of flight (UPLC-QToF) analysis have been performed in a previous study to identify the possible compounds present within the essential oil of *Lippia scaberrima* and *Aspalathus linearis* [16, 22]

**Table 1:** The 50% inhibitory concentration (IC_50_) values of the essential oil of *L. scaberrima*, the ethanolic extract of *L. scaberrima* and their combinations with fermented and unfermented *A. linearis* against various human recombinant CYP enzymes or liver microsomal catalyzed reactions

CYP enzyme	Substrate	Recombinant IC_50_^a^ (mg/L) with 95% confidence intervals	Microsomal IC_50_ (mg/L) with 95% confidence intervals	Sample	PRU
CYP1A1	TFD024	N/A^b^	0.40 (0.31–0.51)	*L. scaberrima* ethanolic extract	
CYP1A2	OCA349	4.10 (2.82–5.95)	4.26 (0.32–8.20)		
CYP1B1	TFD008_1	0.07 (0.06–0.07)	No inhibition^c^		
CYP2A6	coumarin	22.9 (15.4–34.69)	18.37 (9.15–27.6)		119,010
CYP2C19	TFD032	4.19 (2.90–5.48)	No inhibition		
CYP2D6	TFD023	7.65 (4.93–12)	44.93 (34.87–58.44)		
CYP3A4	OCA369	0.41 (0.04–16.28)	25.81 (19.36–32.27)		
CYP1A1	TFD024	N/A	0.49 (0.30–0.80)	*L. scaberrima* extract with unfermented *A. linearis*	
CYP1A2	OCA349	1.62 (0.86–2.95)	0.21 (0.09–0.34)		
CYP1B1	TFD008_1	0.21 (0.17–0.24)	Stimulation^d^		
CYP2A6	coumarin	14.08 (9.47–20.47)	7.66 (6.15–9.17)		
CYP2C19	TFD032	8.79 (4.75–15.58)	Stimulation		N/A
CYP2D6	TFD023	4.19 (3.10–5.73)	6.17 (3.67–8.67)		
CYP3A4	OCA369	No inhibition	3.04 (0.79–5.28)		
CYP1A1	TFD024	N/A	0.40 (0.39–0.48)	*L. scaberrima* extract with fermented *A. linearis*	
CYP1A2	OCA349	2.5 (1.57–3.89)	2.22 (-0.62–5.05)		
CYP1B1	TFD008_1	0.25 (0.19–0.32)	No inhibition		
CYP2A6	coumarin	10.16 (6.84–15.15)	12.37 (10.39–14.35)		N/A
CYP2C19	TFD032	6.96 (3.48–10.44)	2.36 (-1.16–5.83)		
CYP2D6	TFD023	6.96 (4.34–11.05)	18.98 (12.87–25.09)		
CYP3A4	OCA369	No inhibition	12.96 (10.79–15.13)		
CYP1A1	TFD024	N/A	No inhibition	*L. scaberrima* essential oil from leaves and aerial parts	
CYP1A2	OCA349	3.91 (3.51–4.32)	7.65 (-9.90–25.19)		
CYP1B1	TFD008_1	5.93 (0.97–10.90)	1.17 (0.64–1.70)		
CYP2A6	coumarin	0.71 (0.45–0.97)	1.40 (0.87–1.92)		N/A
CYP2C19	TFD032	0.04 (0.01–0.08)	No inhibition		
CYP2D6	TFD023	2.66 (0.80–4.46)	6.43 (2.30–10.56)		
CYP3A4	OCA369	No inhibition	15.85 (3.19–28.51)		

^a^50% inhibition concentration (the concentration at which the sample reduced the metabolism of the CYP substrate by 50%) of recombinant CYP enzymes, placental and liver microsomes

^b^No recombinant CYP1A1 available, CYP1A1 is only available within placental microsomes of tobacco smoking mothers

^c^No inhibition of CYP enzyme at the various concentrations tested

^d^Stimulation of CYP enzyme at the various concentrations tested. The IC_50_ values and the 95% confidence intervals (CI) were determined from the appropriate dose response curves

### Essential oil extraction of L. *scaberrima* through hydro- steam distillation

The stems, leaves and flowers of *L. scaberrima* were collected and hydro-steam distilled to produce an essential oil extract for the current study. With the use of a Clevenger apparatus, the aerial parts were hydro-steam distilled for a period of one and a half hours, after which the essential oil was collected and stored at -20 °C until further use.

### Inhibition of placental CYP1A1, microsomal and recombinant CYP1A2, CYP2A6, CYP3A4, CYP1B1, CYP2C19 and CYP2D6 oxidation by the essential oil of *L. scaberrima*, the ethanolic extract of *L. scaberrima* and their combinations with fermented and unfermented *A. linearis*

The 50% inhibitory concentrations (IC_50_) of the essential oil of *L. scaberrima*, the ethanolic ectract of *L. scaberrima* and their combinations with *A. linearis* for human recombinant CYPs, human liver microsomes and placental microsomes were determined by the method previously described by Juvonen et al. [17], Huuskonen et al. [23] and Crespi et al. [24]. The total volume of the incubation mixtures was carried out in 100 µL of Tris–HCL buffer (pH 7.4) and all black, flat bottom 96-wellplates (Costar Corning Incorporated, Corning, New York). The reaction mixtures for the current study consisted of a 20% NADPH-regenerating system in Tris–HCl buffer (pH 7.4), 10 µM coumarin or a derivatized substrate (TFD024, OCA349, TFD008_1, TFD032, TFD023 or OCA369), prepared microsomal protein (0.010 g/L) or recombinant CYP (5 nM) and essential oil, ethanolic extracts of *L. scaberrima* or their combinations with fermented and unfermented *A. linearis* (3- 500 mg/L). For a full reaction (100%) no essential oil or extracts were added, and for the blank reactions, no substrate or enzyme was added. Stock solutions of the essential oil extract and the ethanolic extract of *L. scaberrima* and their combinations with fermented and unfermented *A. linearis* were made so that the final concentration in the incubation mixtures contained only 1% of the total volume. All plates containing the incubation mixtures were pre-incubated at 37 ºC for 10 min. The enzyme reactions were started once NADPH was added. The standard used during the study was 7-Hydroxycoumarin at a concentration range between 0–10 µM. Fluorescent measurements were done using a Victor^2^ 1420 multilabel plate reader (PerkinElmer Life Sciences Wallac, Turku, Finland) with an excitation wavelength of 405 nm and an emission wavelength of 460 nm. The total fluorescent intensity was monitored every 2 min for a total period of 40 min. The concentration was calculated at various time points, and from there, the oxidation rates and the relative inhibitory activity of the samples tested at different concentrations were also calculated. This data was then entered onto GraphPad Prism 5 (San Diego, CA, USA) and fit to a sigmoidal dose response curve with non-linear regression and the concentration at which the samples reduce the metabolic activity of the specific CYP substrate by 50% (IC_50_) calculated. This calculation was based on the equation of vi/v0 = 1/ (1 + i/IC_50_), in which (vi) is the rate at the specific concentration of the sample, (v0) is the rate without the sample (inhibitor), (IC_50_) is the sample concentration with 50% inhibition and (i) is the sample (inhibitor) concentration.

### Synthesis of gold nanoparticles from *L. scaberrima* and gum arabic

The gold nanoparticles were synthesized from *L. scaberrima,* including gum arabic as a capping agent according to the method of Khoobchandani et al. [25] with slight modifications. Briefly, the synthesis consisted of a stock solution of 18 g/ml of the ethanolic extract of the aerial parts of *L. scaberrima*. One milliliter of the stock solution was added to 17 ml of distilled water to obtain a final concentration of 1 mg/ml. The solution was boiled together for approximately 15 min at 45° C to which 36 mg of gum arabic was added and stirred until completely dissolved. Once the gum arabic was dissolved, 300 μl of HAuCl_2_.3H_2_O (0.1 M) solution was added. The formation of the stabilized gold nanoparticles (*LSAUNP*) was observed by a colour change of the original solution to a deep purple. The gold nanoparticle solution was stirred continuously for an additional 15 min.

### Characterization of synthesized gold nanoparticles (LSAUNPs) from *L. scaberrima*

#### Ultraviolet-visual (UV–Vis) spectroscopy and stability of the synthesized gold nanoparticles

UV absorption (full spectral scan) was used to identify the formation and stability of the *LSAUNP*s through the observed Surface Plasmon Resonance (SPR). The absorption spectra were measured on a Bio-Tek Power-Wave XS multi-well plate reader (A. D. P., Weltevreden Park, South Africa). The data was analyzed using KC junior with a spectral scan that ranged from 450 and 800 nm. *In vitro* stability of the gold nanoparticles was investigated in the presence of 10% NaCl, 100% DMEM and 0.5% bovine serum albumin (BSA) to mimic various physiological conditions and media used throughout the current study after 0, 1, 4, 5 and 7 days at a pH of 4 and 7 respectively. A SPR band at 540 nm will confirm the stability of the gold nanoparticles within all the above media tested.

### Transmission electron microscopy analysis (TEM) of LSAUNP

Transmission Electron Microscopy (TEM) analysis was performed on the gold nanoparticles to investigate the formation of the gold nanoparticles synthesized as well as their approximate size and shape. The nanoparticle solution (5 μl) was loaded onto a carbon coated copper grid, left to dry overnight, and examined using a JEOL JEM-2100F Field Emission Transmission Electron microscope (Akishima, Tokyo, Japan) and the micrographs were captured.

### Fourier transform infrared spectroscopy analysis (FTIR)

The gold nanoparticle solution was freeze-dried under a vacuum at 0.2 atm and -50° C. To investigate the surface functional groups of the gold nanoparticles synthesized, the freeze-dried gold nanoparticles were compressed to form a pellet and examined using the PerkinElmer Spectrum FTIR Spectrometer across the wave range extending from 4000 to 400 cm^−1^.

### X-ray diffraction (XRD)

The previously freeze-dried gold nanoparticles were loaded onto mounting stages utilizing glass cover slips. With a PANalytical X’Pert PRO (PANalytical, Almelo, Netherlands), the analysis of the crystal structure of the synthesized gold nanoparticles was investigated. The analysis was done through the irradiation of the gold nanoparticles with monochromatized Cu K_α_ radiation (λ = 1.54 Å) between 30 and 90° (2θ) and a step size of 0.02°. The settings for the voltage were set at 45 kV and the current at 40 mA.

### Thermogravimetric analysis (TGA)

The thermal decay of the gold nanoparticles was determined using a TG Q5000 V20.13 build 39 (TA instruments, Wilmington, DE, USA). The previously prepared freeze-dried gold nanoparticles (10 mg) were loaded onto a platinum pan and heated from 40 °C to 900 °C with a ramp setting of 30 °C/min.

### Cytotoxicity analysis of the ethanolic extract of *L. scaberrima* and the synthesized gold nanoparticles

Cell viability was determined by the method of Berrington and Lall [26]. Liver hepatocellular carcinoma (HepG2 cells (100 μl) were counted and then seeded into 96 well plates with a cell density of 50 000 cells/ml after which the plates were left to incubate overnight at 37° C with 5% CO_2_ to allow for attachment. The ethanolic extract and gold nanoparticles synthesized were prepared to a stock solution of 2000 μg/ml. Serial dilutions were made with the final test concentrations in the plate ranging from 400- to 1.53 μg/ml. The plates were then further incubated for 72 h at 37° C with 5% CO_2_. The controls used during the current study included a solvent control (DMSO 2%) and a positive control, Actinomycin D, which had a final test concentration of 0.5- 0.002 μg/ml. After 72 h of incubation, PrestoBlue (20 μl) was added to each well, and the plates were further incubated for 4 h to allow for a colour change to occur. Finally, the absorbance was read at 490 nm with a reference wavelength of 690 nm using a BIO-TEK Power-Wave XS multi-well reader. The assay was performed in triplicate, and the mean IC_50_ values were calculated using GraphPad Prism 4.

## Results

### Inhibition of human placental CYP1A1, microsomal and recombinant CYP1A2, CYP2A6, CYP3A4, CYP1B1, CYP2C19 and CYP2D6 oxidation by the essential oil of *L. scaberrima*, the ethanolic extract of *L. scaberrima* and their combinations with fermented and unfermented *A. linearis*

To determine if the essential oil of *L. scaberrima*, the ethanolic extract of *L. scaberrima*, and their combinations with *A. linearis* could inhibit individual human CYP enzymes, concentration dependent inhibitions were determined for eight recombinant CYP enzymes and selective CYP substrates including hepatic and placental microsomes (Table 1). The inhibition potency of the extracts varied against the different CYP enzymes tested. The greatest inhibition was observed for CYP2C19 by the essential oil with an IC_50_ value of 0.04 mg/L (0.01–0.08) as well as CYP1B1 by *L. scaberrima* with an IC_50_ value of 0.07 mg/L (CI: 0.06–0.07). The most potent inhibition across all CYP enzymes tested took place against recombinant CYP1B1, with IC_50_ values that were less than 1 mg/L for all extracts tested except the essential oil. Although no significant inhibition is noted for human placenta microsomal CYP1A1, the values were slightly lower, in relation to the IC_50_ values obtained for the rest of the recombinant and microsomal CYP enzymes tested.

The IC_50_ value range of the tested essential oil, extracts and combinations were found to be between 1 – 10 mg/L for both recombinant CYP1A2 and CYP2D6. Other recombinant CYP inhibitory IC_50_ values were higher than 10 mg/L and, therefore, showed no noteworthy inhibition. Most of the potent and moderate enzyme inhibition of the CYP enzymes investigated resulted from the combination of *L. scaberrima* and unfermented *A. linearis*.

### Characterization of synthesized gold nanoparticles (LSAUNPs) from *L. scaberrima*

#### Ultraviolet-visual (UV–Vis) spectroscopy and stability of the synthesized gold nanoparticles

To investigate the formation of the gold nanoparticles from the *L. scaberrima* extract, the UV–Vis absorption spectra experiment of the mixture was investigated [27]. A strong absorbance band can be found in the visible region of between 500–600 nm. The λ_max_ was found at approximately 540- 555 nm for all the days measured (Day 0–7) (Supplementary data Figure S1-6). The stability of the gold nanoparticles within several different media relevant to the current study (NaCl, BSA and DMEM) over a certain period (0, 1, 4, 5, 7 days) and pH (pH 7 and pH 9) were investigated through their UV–Vis absorption spectra. For all the sample ratios (2:3, 1:4 and 1:9), the different pH’s and the different media tested, the peak absorbance for the synthesized gold nanoparticles was at 540 nm.

### Transmission electron microscopy analysis of LSAUNP

**Fig. 1:**
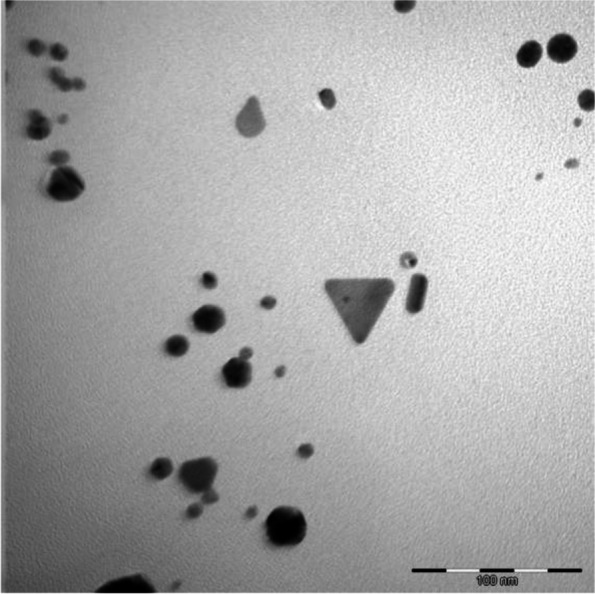
Several different shapes and sizes of gold nanoparticles formed from *L. scaberrima* and gum arabic as the capping agent investigated using TEM microscopy

Gold nanoparticles, mainly spherical, were obtained, as shown in Fig. 1. The synthesized gold nanoparticles were found to be present in several different sizes and predominantly in a spherical shape but were also triangularly shaped.

### Fourier transform infrared spectroscopy analysis (FTIR)

**Fig. 2:**
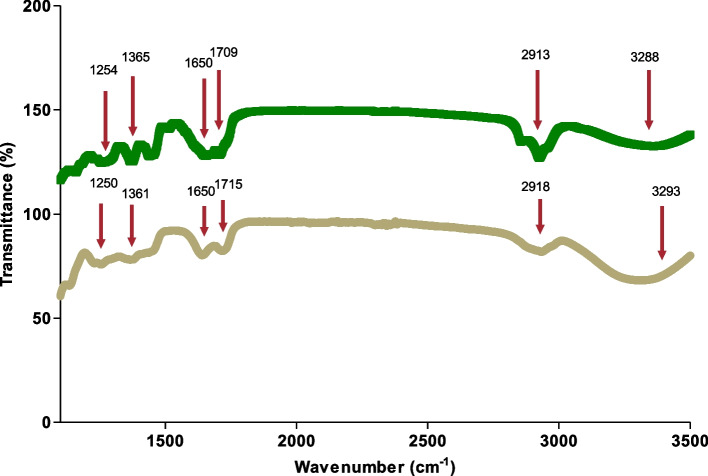
FTIR measurement of the gold nanoparticles synthesized and the ethanolic extract of *L. scaberrima* showing several functional groups present at their specific wavenumbers (cm^−1^) (indicated by the arrows)

The FTIR measurements were conducted to investigate the possible molecules responsible for the capping and stabilization of the gold nanoparticles synthesized when the ethanolic extract of *L. scaberrima* was added, as shown in Fig. 2. The peaks found in the FTIR spectra represent the functional groups that might be present on the surface of the gold nanoparticles when synthesized. The FTIR of the ethanolic extract of *L. scaberrima* (blue line) and the gold nanoparticles synthesized (*LSAUNP*) (green line) showed broad peaks at 3288 cm^−1^ and 3293 cm^−1^ characteristics of O–H bonds contributed to by the carboxylic acids. The peaks observed at 2913 cm^−1^ and 2918 cm^−1^ were attributed to the C-H bonds of the alkenes. The very weak peaks at 2344- and 2343 cm^−1^ are due to C = O groups, whereas the peaks at 2295 cm^−1^ and 2298 cm^−1^ are due to the C = C bands (alkenes). The peaks that occur in the range of 1750- 1650 cm^−1^ and 1715- and 1650 cm^−1^ are found due to the presence of carbonyls (C = O), while the peaks depicted in the ranges of 1365- 1254 cm^−1^ and 1361–1250 cm^−1^ are due to nitrile compounds (N–O). All the peaks that were found in the spectra of the crude extract corresponded to the peaks found in the gold nanoparticles synthesized. These peaks are all due to the functional groups already present in the ethanolic extract of *L. scaberrima.*

### X-ray diffraction (XRD)

**Fig. 3:**
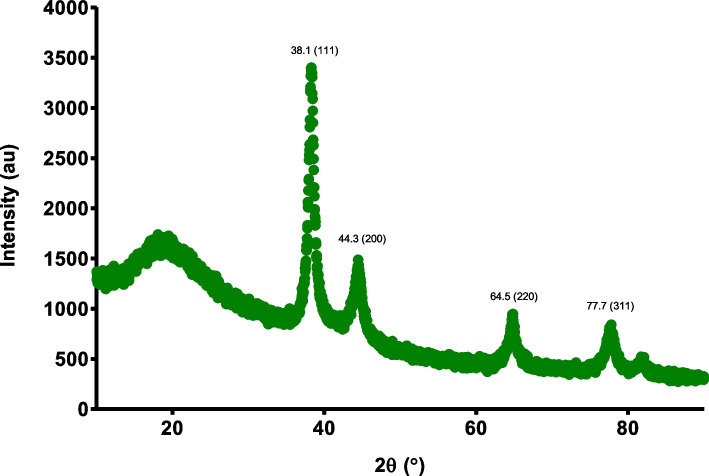
The XRD pattern of the synthesized gold nanoparticles from *L. scaberrima* and gum arabic as a capping agent

The gold nanoparticles synthesized were subject to XRD analysis to confirm the formation of the gold nanoparticles as well as the confirmation of the crystalline structure of the gold present. Figure 3 shows the XRD pattern obtained from gold nanoparticles synthesized from the *L. scaberrima* extract and the capping agent, gum arabic. The intense peak and planes of the gold nanoparticles found at 38.2° (111), 44.3° (200), 64.5° (220) and 77.7° (311) signifying the presence of gold nanoparticles that are crystalline. The planes are also representative of the face centered cubic (fcc) planes of gold (Au). The most distinctive peak was found at 38.2° and the (111) plane.

### Thermogravimetric analysis (TGA)

**Fig. 4:**
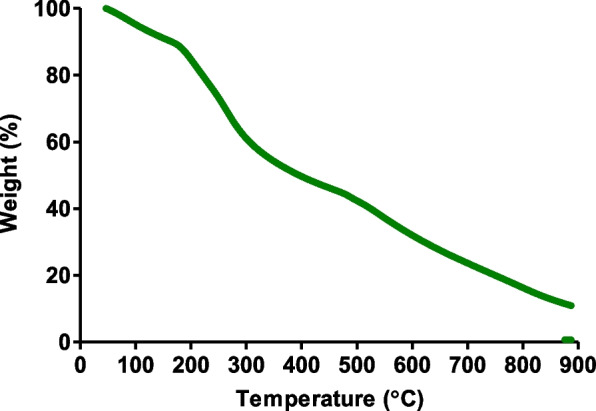
Thermogravimetric analysis (TGA) of the gold nanoparticles synthesized from *L. scaberrima* extract and gum arabic as a capping agent recorded between 40 °C and 900 °C

The TGA spectra showed significant weight loss (%) between 46- and 900 ˚C (Fig. 4). This reduction in weight (%) decreased linearly with the increase in temperature. This decrease indicated that at higher temperatures, the compounds from *L. scaberrima* that surround the gold nanoparticles, acting as the capping and stabilizing components for the nanoparticles, were completely degraded. Hence, this serves as confirmation that the gold nanoparticles synthesized from *L. scaberrima* and gum arabic were capped with organic compounds.

### Cytotoxicity analysis of the ethanolic extract of *L. scaberrima* and the synthesized gold nanoparticles

Previous studies conducted by Reid et al. [16] indicated that the ethanolic extract of *L. scaberrima* tested against liver hepatocellular carcinoma (HepG2) cells had an IC_50_ value of 109.20 ± 8.05 μg/mL after 72 h of incubation (Table 2). The synthesized gold nanoparticles (*LSAUNP*) tested against HepG2 cells in the current study had an IC_50_ value of 268.50 ± 1.34 μg/mL. It has been observed that the IC_50_ value increased twofold when compared to the ethanolic extract of *L. scaberrima*.

**Table 2:** The cytotoxicity (IC_50_) values of the ethanolic extract of *L. scaberrima* and the synthesized gold nanoparticles of *L. scaberrima* and gum arabic (*LSAUNP*)

Sample	HepG2 IC_50_^a^ (μg/mL) ± SD 72 h
*L. scaberrima*^*b*^	109.20 ± 8.05
*LSAUNP*^*c*^	268.50 ± 1.34
Actinomycin-D^d^	8.56 ± 8.24

^a^50% inhibitory concentrations

^b^Determined from a previous study by the authors (Reid et al., 2020)

^c^Gold nanoparticles synthesized from *L. scaberrima* extract and gum Arabic

^d^Positive control for HepG2 cells

## Discussion

### Inhibition of placental CYP1A1, microsomal and recombinant CYP1A2, CYP2A6, CYP3A4, CYP1B1, CYP2C19 and CYP2D6 oxidation by the essential oil of *L. scaberrima*, the ethanolic extract of *L. scaberrima* and their combinations with fermented and unfermented *A. linearis*

Metabolism is an important component of the kinetic characteristics of herbal constituents, and it often determines the internal dose and concentration of these effective constituents at the target site. *In vitro* analysis involving liver enzymes is a frequently used method to determine the possibility of *in vivo* drug interactions and has yielded dependable results thus far [28]. Liver microsomes contain several CYP enzymes together and no specific substrates are then oxidized by several CYPs as compared to single recombinant enzyme catalyzing this specific substrate. Therefore, an effect is more efficiently seen within the recombinant proteins as compared to the microsomes, which include a variety of enzymes and often a somewhat higher level of substrates.

Varied levels of inhibition were observed for the essential oil, the ethanolic extract of *L. scaberrima* and their combinations with fermented and unfermented *A. linearis*. The essential oil had the greatest inhibition of CYP2C19 with an IC_50_ value of 0.04 mg/L (0.01–0.08). This inhibition of CYP2C19 indicated targeted specificity by the essential oil as no other activity was detected. CYP2C19 is responsible for the metabolism of many important drugs, such as proton pump inhibitors and anti-depressants [29]. A marked decrease in the activity of this enzyme might cause metabolic interactions of these important drugs, and therefore, a detailed investigation needs to be done on which constituents might be responsible for the inhibitory activity found.

The ethanolic extract of *L. scaberrima* had the highest inhibitory activity against CYP1B1 with an IC_50_ value of 0.07 mg/L (CI: 0.06–0.07). CYP1B1 is known to be extrahepatic and a key CYP enzyme involved in the metabolism of hormone responsive cancer. CYP1B1 is also present within the liver albeit in lower levels and is found to be responsible for the oxidative metabolism of estrogen and the conversion of certain compounds from procarcinogens to carcinogens [30, 31]. Due to inhibition by all the extracts and the combinations tested against CYP1B1, this warrants further investigation into the mechanisms of the *in vitro* and *in vivo* inhibition mediated by these samples. Although no significant inhibition of microsomal CYP1A1 was found, it is important to note that specificity is key when considering the inhibition of CYP1B1 and CYP1A1, as the inhibition of CYP1B1 could be a main target for cancer prevention, whereas CYP1A1 plays a key part in metabolically activating dietary compounds that aid in cancer prevention as well as detoxifying procarcinogens found in the environment, although CYP1A1 has also been implicated in liver carcinogenesis through BaP metabolic activation [32, 33]. *Lippia scaberima* and *A. linearis* are both recognized for their high flavonoid content. Flavonoids are known for their interactive potential with CYP1 as they may act as inhibitors and substrates [34]. Furthermore, flavonoids have been implicated several times as the main component exerting inhibition of CYP enzymes [2]. A study conducted by Patel et al. [35] indicated that aspalathin, an abundant compound found within *A. linearis*, together with fermented and unfermented *A. linearis* showed inhibition of CYP3A4 when compared to erythromycin at set concentrations of 25, 50 and 100 μg/mL. Structure-based molecular docking studies and computer modelling could identify the potential binding sites and whether the compounds found within these extracts bind on the active centre or allosteric sites. This should give more insight into the possible mechanisms of actions involved.

### Characterization of synthesized gold nanoparticles (LSAUNPs) from *L. scaberrima*

#### Ultraviolet–visible (UV–Vis) spectroscopy and stability of the synthesized gold nanoparticles

To investigate the formation of the gold nanoparticles from the ethanolic extract of *L. scaberrima*, the full absorption spectrum of the mixture was investigated [27]. The results observed corresponded well with previous findings in the literature. In another study, the synthesis of gold nanoparticles was done using extracts of *Crocus sativus* and showed a corresponding absorbance value at 549 nm [36]. A similar study conducted by Elia et al. [37] investigated the potential for the synthesis of gold nanoparticles using four common household plants and observed their absorbance values similarly at 549 nm. The method used in the current study for synthesizing gold nanoparticles from ethanolic extracts, including the use of gum arabic as a capping agent, is therefore validated and may be a method that can be followed by other researchers and institutions as it has an easy setup and requires a minimal amount of time to synthesize.

### Transmission electron microscopy analysis (TEM) of LSAUNP

The observations made using TEM analysis were mostly of spherically shaped gold nanoparticles, although there were several different sizes present, including nanoparticles that were triangular. The variety of sizes and shapes have been described before in the literature and is a common occurrence with gold nanoparticles [38].

### Fourier transform infrared spectroscopy analysis (FTIR)

The FTIR analysis of the synthesized gold nanoparticles featured various peaks that were also found in the ethanolic extract of *L. scaberrima*, confirming the presence of these secondary compounds on the surface of the gold nanoparticles synthesized. These secondary compounds act as capping agents on the surface of the gold nanoparticles which assist in their stability but also to prevent agglomeration from occurring. Limonene forms part of the alkene functional group (C = C) and due to its presence within the ethanolic extract of *L. scaberrima*, it is, therefore, not uncommon to find it on the surface of the newly formed nanoparticles. The *Lippia* genus is also known for the presence of a wide array of flavonoids and phenolics [38]. Many different compounds, including flavonoids and diterpenoids, as well as polysaccharides are known for the reduction of gold nanoparticles. These compounds are known to be present within *L. scaberrima* [39]. The FTIR analysis confirmed that the reduction of the Au^3+^ to Au^0^ were due to the capping material found within the plant extract and the gum arabic. Proteins that are found within the plant extract may also interact and bind to the gold nanoparticles through either free amino or carboxyl groups found within the proteins.

### X-ray diffraction (XRD)

The gold nanoparticles were subject to XRD analysis to confirm the formation of the gold nanoparticles as well as the confirmation of the crystalline structure. The fcc of the synthesized gold nanoparticles matched those of the Joint Committee on Powder Diffraction Standards (JCPDS no. 00–004-0784), which confirms the presence of crystalline gold. Sett et al. [40] also reported that the peaks of gold nanoparticles formed will be in this region currently seen in the study and that this is a typical XRD pattern for the formation of gold nanoparticles.

### Thermogravimetric analysis (TGA)

The decrease in weight (%) that was observed from the TGA spectra indicated that at higher temperatures, the compounds from *L. scaberrima* that are surrounding the gold nanoparticles, acting as the capping and stabilizing components for the nanoparticles, were completely degraded. Hence, this served as a confirmation that the gold nanoparticles synthesized from *L. scaberrima* were capped with organic compounds and were stable at most of the set temperatures across various time points until completely degraded.

### Cytotoxicity analysis of the ethanolic extract of *L. scaberrima* and the synthesized gold nanoparticles

Previous studies conducted by Reid et al. [16] indicated that the ethanolic extract of *L. scaberrima* tested against HepG2 cells had a much lower IC_50_ value when compared with the synthesized gold nanoparticles. This may in part be due to the functional groups found to be present on the surface of the nanoparticles. The toxicity of nanoparticles, especially that of gold nanoparticles has been shown to be linked to size, where gold nanoparticles that were tested between 8 and 37 nm size have had devastating results on the viability of the mice studied [41]. All other sizes up to 100 nm that were tested during this study had no lethal effects. The size of the synthesized gold nanoparticles, therefore, is shown to have a dramatic effect on its toxicity potential and should be further investigated. *Lippia javanica* is a well-known medicinal plant found within the same genus and is also indigenous to South Africa. Piperitenone, a constituent within the extract and the essential oil has been shown to have a low toxicity profile when tested against human adenocarcinoma (HCT- 8) cells with an IC_50_ value of 265.6 ± 8.53 μg/mL. Similar results were found for limonene, the major constituent within the essential oil extract, which had an IC_50_ value of 285 ± 49 μM (38.83 μg/mL) against human lung (CCD-19Lu) cells after 24 h of incubation [42]. A carvone-rich chemotype of *Lippia alba* rich in limonene and carvone showed slight toxicity against the human cervix epithelioid carcinoma (HeLa) cell line with a CC_50_ (cytotoxicity concentration) value of 74.5 ± 13.1 μg/mL and a CC_50_ concentration value against African green monkey kidney (Vero) cells of more than 200 μg/mL, the highest concentration tested. Furthermore, individual compounds of limonene and carvone were tested against the HeLa and Vero cell lines and found not to be cytotoxic [43]. L-carvone showed considerable toxicity towards breast cancer cell lines and was explored for its apoptotic ability within these cell lines. The 50% inhibitory concentration (IC_50_) values against MCF-7, MDA MB 231 and MCF 10 A were 180.26 μg/mL (1.2 mM), 150. 22 μg/mL (1.0 mM) and 3004, 4 μg/mL (20 mM), respectively [44]. It has therefore been confirmed that the synthesized gold nanoparticles of *L. scaberrima* when compared to the ethanolic extract of *L. scaberrima* decreased the toxicity potential by approximately twofold when tested in the current study. This seems to be linked to the specific functional groups that are present on the surface of the nanoparticles and the actual size of the nanoparticle. An in-depth analysis on the toxicity profile of various other cancerous and non-cancerous cells can be investigated to identify a possible inhibitory mechanism of action and any trends (cancerous vs. non-cancerous). Further analysis of different capping agents that can be used, including biotransformation (fermentation) to enhance and modify existing compounds found with the plant extract, may also be investigated. If the mechanisms by which the cellular toxicity profile is lowered can be determined, these methods can be incorporated into many previously investigated plants that were deemed cytotoxic.

## Conclusion and future prospects

The present study shows that many herb-drug CYP-interactions are possible with the essential oil of *L. scaberrima*, the ethanolic extracts of *L. scaberrima* and their combinations with fermented and unfermented *A. linearis* albeit at significantly lower levels. This further supports that the pharmacokinetic profile of a herbal mixture and an active pure compound isolated from medicinal plants are indeed important and necessary for the prediction of possible herbal-drug interactions. As previously mentioned, many medicinal plants are being used adjunctly and it is important to determine if there is any herb-drug interaction potential. It can, however, be concluded that *L. scaberrima* and *A. linearis* can be used as an adjuvant due to only low levels of interactions with the hepatic or extrahepatic CYP enzymes.

The reduction of the gold by using *L. scaberrima* and gum arabic, is a reliable and economic method of obtaining gold nanoparticles with a lowered cytotoxicity potential as can be seen in the current study. Many methods have been proposed for the synthesis of gold nanoparticles and includes the addition of plant extracts that already act as natural stabilizers. In the present study, most of the compounds found within *L. scaberrima* are known for their antioxidative properties and are thought to be the main reducing agents that led to the swift formation of the gold nanoparticles. Many of these antioxidants are highly water soluble and due to this property, may act as a better reducing agent for the synthesis of gold nanoparticles [37]. Further investigation is, however, required into the size distribution of the nanoparticles as well as the effect of gum arabic on the activity of the synthesized gold nanoparticles.

In South Africa, especially with our rich biodiversity and traditional knowledge on infections and diseases treatments, a rich source of untapped information exists, which may hold the key for treating diseases such as tuberculosis. The successful synthesis of gold nanoparticles has been shown to be possible with the use of plants, further highlighting the potential of this resource, which may introduce several novel compounds and extracts for developing drugs from natural products.

It is important to note that the calculation of an IC_50_ value in the CYP inhibition assays do not have the ability to distinguish whether the lead compound in the extract or the extract itself causes inactivation of the enzyme or whether a reversible inhibition of the enzyme occurred. Further it is also not able to determine the exact kinetic mechanism involved if indeed it is reversible inhibition. Although the IC_50_ values can indicate the potential of the inhibition of the extract further analysis is required. This includes analysis such as identification of the lead compounds and performing molecular modelling to determine the possible mechanism of inhibition, although as previously mentioned direct inhibition might not be applicable as reversible inhibition or direct inhibition of the enzyme might have taken place. Further investigation into these direct mechanisms needs to be conducted to determine the mechanism of inhibition of the compound/extract of interest.

### Supplementary Information

**Supplementary Material 1.**

## Data Availability

The datasets used and/or analysed during the current study are available from the corresponding author on reasonable request.

## Abbreviations

CYP P450: Cytochrome P450
IC_50_: 50% Inhibitory concentration
GC–MS: Gas chromatography-mass spectrometry
UPLC-QToF: Ultra-performance liquid chromatography: quantitative time of flight
UV–Vis: Ultraviolet-visual
LSAUNP: Gold nanoparticles synthesized from *Lippia scaberrima*
TEM: Transmission Electron microscopy
FTIR: Fourier Transform Infrared Spectroscopy
XRD: X-ray diffraction
TGA: Thermogravimetric analysis
HepG2: Liver hepatocellular carcinoma cells
