# Supplementary material for: Evaluation of Lippia scaberrima Sond. and Aspalathus linearis (Burm.f.) R. Dahlgren extracts on human CYP enzymes and gold nanoparticle synthesis: implications for drug metabolism and cytotoxicity
Source: BMC Complement Med Ther. 2024 Apr 5;24:152. doi: 10.1186/s12906-024-04439-9 (PMC10996199; doi:10.1186/s12906-024-04439-9)
Supplement: Supplementary file 1 — Supplementary Material 1. [file 12906_2024_4439_MOESM1_ESM.docx]

Supplementary data

Figure S1: The stability of gold nanoparticles in NaCl at pH 7 at various ratios (2:3, 1:4 and 1:9)

Figure S2: The stability of gold nanoparticles in BSA at pH 7 at various ratios (2:3, 1:4 and 1:9)

Figure S3: The stability of gold nanoparticles in DMEM at pH 7 at various ratios (2:3, 1:4 and 1:9)

Figure S4: The stability of gold nanoparticles in NaCl at pH 9 at various ratios (2:3, 1:4 and 1:9)

Figure S5: The stability of gold nanoparticles in BSA at pH 9 at various ratios (2:3, 1:4 and 1:9)

Figure S6: The stability of gold nanoparticles in DMEM at pH 9 at various ratios (2:3, 1:4 and 1:9)
